# Combined *ex vivo* and *in vivo* evaluation of dolutegravir embryotoxicity: NTDs and yolk sac vascular abnormalities: Dolutegravir induces NTDs and YS abnormalities

**DOI:** 10.3724/abbs.2025142

**Published:** 2025-11-03

**Authors:** Ruifang Ao, Ran Li, Zelin Li, Guicai Wu, Haoran Xu, Xuecong Wang, Jiayi Du, Xiaozheng Zhang, Jun Xie

**Affiliations:** MOE Key Laboratory of Coal Environmental Pathogenicity and Prevention Shanxi Key Laboratory of Birth Defect and Cell Regeneration Department of Biochemistry and Molecular Biology Shanxi Medical University Taiyuan 030001 China

**Keywords:** DTG, whole embryo culture, yolk sac blood circulation, NTD, oxidative stress

## Abstract

Dolutegravir (DTG) disrupts mouse embryonic development in a dose-dependent manner, culminating in neural-tube defects (NTDs). Using whole embryo culture (WEC), mouse embryos at embryonic day 8.5 (E8.5) are cultured for 24–48 h with 8, 10, or 12 μM DTG. The results reveal that higher DTG concentrations dose-dependently disrupt yolk sac development and markedly increase the frequency of NTDs. *In vivo* NTD models are generated by intraperitoneally injecting DTG at a dose of 7.5 mg/kg, and the resulting embryos exhibit disrupted yolk sac blood circulation, embryonic growth restriction, and malformations. Mechanistic studies suggest that DTG contributes to NTDs by inducing apoptosis: DTG exposure activates the Nrf2-SOD1/CAT antioxidant axis, yet it culminates in increased apoptosis and suppressed proliferation, ultimately impairing yolksac vasculogenesis and neuralepithelial closure, thereby producing NTDs. This study provides new evidence for assessing the potential risk of DTG in embryonic development and highlights the need to re-evaluate its clinical safety in future applications.

## Introduction

Neural tube defects (NTDs) are the second most common structural birth defects after congenital heart defects, and they impose a significant burden on both society and families [ 1, 2] . The occurrence of NTDs is attributed to a combination of genetic and environmental factors [3]. Dolutegravir (DTG) is a non-nucleoside reverse transcriptase inhibitor that effectively blocks the integration of HIV DNA into the host cell genome [4]. Notably, DTG can cross the placenta and enter fetal circulation, thereby providing protection prior to fetal exposure [5]. Owing to its robust resistance barrier and rapid viral suppression, DTG has been adopted as the preferred first-line antiretroviral treatment (ART) in low- and middle-income countries, including in pregnant women, since its initial FDA approval in 2013 [6].

However, a birth surveillance study in Botswana in 2018 revealed that women who began DTG treatment at conception had a significantly elevated incidence of NTDs in their infants compared with those receiving other antiretroviral drugs [ 7– 9] . The rate of NTDs in infants from DTG-treated mothers was found to be eight times higher than that of other ARVs. Although the 2019 data indicated a reduction in NTD incidence among DTG-exposed infants [10], the rate remained slightly higher than that of other ART drugs. Studies by Mmakgomo *et al*. [11], Robert *et al*. [12] and others [ 13– 16] confirmed DTG’s association with NTDs, whereas researches by Stanislaus *et al*. [17] and Lorraine *et al*. [18] suggested no embryotoxicity. Given the essential role of DTG as a potent antiretroviral, further animal model studies are needed to evaluate its potential link to NTDs and mitigate any adverse effects [ 9, 19] . DTG exposure has been shown to increase reactive oxygen species, triggering oxidative stress that threatens normal embryonic development [ 20, 21] . In response, the Nrf2 (nuclear factor erythroid 2-related factor) signaling pathway is activated, inducing the expression of antioxidant genes such as superoxide dismutase 1 ( *SOD1*) and catalase ( *CAT*) to restore redox balance [22]. However, when Nrf2 activation alone is insufficient to counteract oxidative damage, disrupted cell proliferation and excessive apoptosis may occur [22]. Dysregulation of these processes has been strongly implicated in the pathogenesis of NTDs under oxidative stress conditions [ 23, 24] . Furthermore, previous studies confirmed elevated apoptosis in the neural tissues of both human and mouse NTD patients [ 25, 26] . These findings raise critical concerns about whether DTG disrupts early developmental processes and contributes to NTD formation.

In both humans and mice, the post-implantation stage is critical for organogenesis. Neural tube closure begins around embryonic day (E) 8.0 and is completed by E10.0 in mice, corresponding to gestational weeks 3--4 in humans [ 27, 28] . Failure of this process results in NTDs such as spina bifida and anencephaly. During this period, the yolk sac also plays an essential role in supporting nutrient uptake, hematopoiesis, and gas exchange before placental function is established [29]. Yolk sac vasculogenesis occurs in parallel with neural tube formation [30]. Thus, both processes are closely synchronized and vulnerable to disruption, which may lead to embryonic lethality or malformations.

Determining the critical dose is vital when assessing the effects of DTG on embryonic neural tube development, as embryonic development is highly sensitive to dosage variations. WEC (whole embryo culture) technology closely simulates mammalian embryonic development *in vitro*, maintains the same developmental pace as in utero, and avoids maternal and placental interference [ 31– 33] . Thus, WEC offers a valuable model for evaluating DTG embryotoxicity.

In this study, we investigated the effects of DTG on mouse embryo development using the WEC system to determine safe DTG blood concentrations during the neural tube closure period and validated its embryotoxicity *in vivo*. By analyzing oxidative stress markers, gene expression, and apoptosis levels in NTD embryos, the potential mechanisms of DTG-induced NTDs were explored. Our findings provide scientific evidence for the safe use of DTG in pregnant women during the perinatal period, thereby contributing to the prevention of adverse pregnancy outcomes.

## Materials and Methods

### Animal husbandry

All procedures were conducted in accordance with the policies for animal care, welfare, and treatment at Shanxi Medical University and were approved by the Institutional Animal Care and Use Committee (IACUC). Six-week-old female specific-pathogen-free (SPF) Institute of Cancer Research (ICR) mice were purchased from Beijing Vital River Laboratory Animal Technology Co., Ltd. (SCXK Beijing 2021-0006, No. 110011241103281113; Beijing, China). Mated females were individually housed in plastic cages with bedding material in a controlled environment (64–79 F, 30%–70% relative humidity) with a 12/12-h light/dark cycle. Food (Certified Rodent Die, #5001; PMI Nutrition International, Brentwood, USA) and filtered tap water were provided. The vaginal plugs were checked the following morning, and the day of detection was designated E0.5.

### WEC *in vitro*

Mouse embryos were cultured following the established protocol [ 31– 33] . Pregnant female ICR mice were euthanized at midday on E8.5, and embryos were harvested for subsequent experiments (Whole Embryo Rotation Culture System Research Edition; WEC001-S; Beijing LiangYi Biotechnology Co., Ltd., Beijing, China). Under a stereomicroscope (Leica M165FC; Leica, Wetzlar, Germany), Reichert’s membrane was carefully removed, and embryos at the appropriate developmental stage (4–5 somite pairs) were selected for the study. The selected embryos were placed into pre-warmed sterile bottles containing culture medium composed of 80% heat-inactivated rat serum (1 mL per embryo), 20% 0.9% NaCl solution, 10 μg/mL penicillin-streptomycin, 2 mg/mL glucose, and either 0.04% DMSO (control group) or DTG at final concentrations of 8, 10, or 12 μM. The culture conditions were maintained at 37°C with a rotation speed of 30 rpm. The initial gas mixture was set to 5% O _2_, 5% CO _2_, and 90% N _2_, and after 10 h, it was adjusted to 20% O _2_, 5% CO _2_, and 75% N _2_ [ 31– 33] . The culture medium was replaced every 24 h. Each group ultimately contained at least 16 embryos, with representation from each litter to ensure balanced distribution among groups. Photographs were taken every 24 h, and the embryos were cultured for a total of 48 h. Embryo development was assessed by analyzing morphological characteristics, crown-rump length, neural tube closure in both the head and tail regions, yolk sac diameter, and the establishment of blood circulation to evaluate the effects of DTG on embryogenesis.

### *In vivo* DTG-induced NTD mouse model

To establish a DTG-induced NTD mouse model, pregnant ICR mice at E7.5 were randomly divided into control and experimental groups. The experimental group was further divided into three subgroups: those receiving intraperitoneal injections of saline-DMSO buffer (control), those receiving 7.5 mg/kg DTG, or those receiving 15 mg/kg DTG. Injections were administered once daily from E7.5 to E9.5. On E10.5, embryonic development was analyzed under a stereomicroscope (Leica M165FC) to evaluate developmental morphology and neural tube closure. For further analysis of neural tube closure, additional groups of pregnant mice received daily intraperitoneal injections of 7.5 mg/kg DTG or an equivalent volume of saline-DMSO buffer from E7.5 to E10.5. Embryonic brain tissues and yolk sacs were collected at E10.5 for structural analysis and mechanistic studies.

### ROS detection in embryos

E10.5 embryonic heads from the control and DTG-treated groups were separately dissociated into cells and suspended in 200 μL of PBS. The cells were incubated with 1 μL of DCFH-DA (Nanjing Jiancheng Bioengineering Institute, Nanjing, China) at room temperature in the dark for 30 min. After incubation, the cells were washed with PBS and centrifuged (1600 *g*, 5 min), stained with DAPI for 10 min, washed again, and mounted on glass slides. The fluorescence signals were observed under a fluorescence microscope (480 nm for ROS and 360 nm for nuclei). The green fluorescence intensity was quantified using ImageJ.

### Hematoxylin and eosin (HE) and immunohistochemistry (IHC) analysis

Fetal brain and yolk sac samples were fixed in 4% neutral formaldehyde overnight, embedded in paraffin, and sectioned at 4 μm. The sections were deparaffinized in xylene, rehydrated through a graded ethanol series, and rinsed in water. Tissue morphology was assessed via HE staining.

For IHC analysis, antigen retrieval and blocking were performed before incubation with primary antibodies against P-Nrf2 (1:800, #ET1608-28; Huabio, Hangzhou, China), SOD1 (1:300, 10269-1-AP; Proteintech, Wuhan, China), catalase (CAT, 1:500, 21260-1-AP; Proteintech), PCNA (1:800, ab92552; Abcam, Cambridge, UK), CC3 (1:800, #9664; Cell Signaling Technology, Danvers, USA), and CD31 (1:500, ab28364; Abcam) overnight. The sections were then incubated with goat anti-rabbit IgG secondary antibody conjugated to horseradish peroxidase (HRP) (1:500 in TBS-T buffer) for 2 h at room temperature. Positive signals were detected via a DAB kit (Zhongshan Golden Bridge, Beijing, China), followed by hematoxylin counterstaining. The IHC procedure followed a well-established protocol [34]. Images were captured with a PA53 biological microscope (Motic, Fuzhou, China).

### TUNEL assay

Apoptosis was assessed using the Cell Death Detection kit (POD; Roche, Basel, Switzerland). E11.5 sections were washed in PBS, fixed in 4% paraformaldehyde (30 min), and treated with 3% H _2_O _2_ in methanol (20 min, dark). The samples were incubated with a TdT/dUTP mixture (1:9) at 37°C for 1 h and converter-POD for 30 min, counterstained with DAPI and viewed under a FV1000 laser scanning confocal microscope (Olympus, Tokyo, Japan) [34].

### PCNA and CC3 labelling and immunofluorescence staining

For PCNA and CC3 staining [34], fetal brains and yolk sacs were fixed in 4% formaldehyde, paraffin-embedded, and sectioned (4 μm). The sections were deparaffinized, rehydrated, washed, subjected to antigen retrieval in pH 6.0 sodium citrate buffer and blocked with 10% donkey serum at 37°C for 1 h. The samples were subsequently incubated with primary antibodies against PCNA (1:800, ab92552; Abcam) and CC3 (1:800, #9664; Cell Signaling Technology) overnight at 4°C, followed by incubation with a fluorescent secondary antibody for 40 min at room temperature and DAPI counterstaining (containing an anti-fluorescence quenching sealant, BL739A; Biosharp, Hefei, China). Confocal images (FV1000) were captured and analyzed using ImageJ.

### Real-time PCR

Total RNA from E11.5 embryos was extracted with TRIzol (15596026; Invitrogen, Waltham, USA) and reverse-transcribed using a PrimeScript kit (R223-01; Vazyme, Nanjing, China). Real-time PCR was then run on a QuantStudio 5 system (ABI, Waltham, USA) with SYBR Premix Ex Taq (Q711-02; Vazyme) under the following conditions: 95°C for 30 s, 95°C for 10 s, and 60°C for 30 s (40 cycles), followed by melting curve analysis. The data were normalized to those of *β-actin*, and the sequences of primers are shown in Supplementary Table S1.

### Western blot analysis

Western blot analysis was carried out as described previously [34]. Briefly, fetal brain proteins were extracted with RIPA buffer on ice and standardized, and 10 μg was separated by 10% SDS-PAGE. After being transferred to PVDF membranes, the membranes were blocked with 5% skim milk, incubated with primary antibodies (anti-SOD, 1:1200, 10269-1-AP, Proteintech; anti-CAT, 1:2000, 21260-1-AP, Proteintech; anti-PCNA, 1:5000, ab92552, Abcam; and anti-CC3, 1:5000, ab214430, Abcam), and then with HRP-conjugated secondary antibodies (1:5000) for 2 h. Signal detection was performed via an enhanced chemiluminescence (ECL) kit (Millipore, Billerica, USA). The experiments were repeated at least three times, with protein bands quantified via ImageJ and with GAPDH and β-actin used as loading controls.

### Statistical analysis

At least three pregnant mice were included in each group. The experimental results were analyzed and visualized using SPSS 26.0, GraphPad Prism 9.5 and ImageJ software. Statistical significance between the experimental and control groups was determined using Student’s *t* test, the Mann-Whitney U test (yolk sac score), and ANOVA with a post hoc Dunnett test (yolk sac diameter, embryonic crown-rump length), with a threshold of *P*  < 0.05 considered statistically significant.

## Results

### DTG inhibits neural tube development and yolk sac circulation in mouse embryos in a dose-dependent manner via the WEC

**Figure 1:**
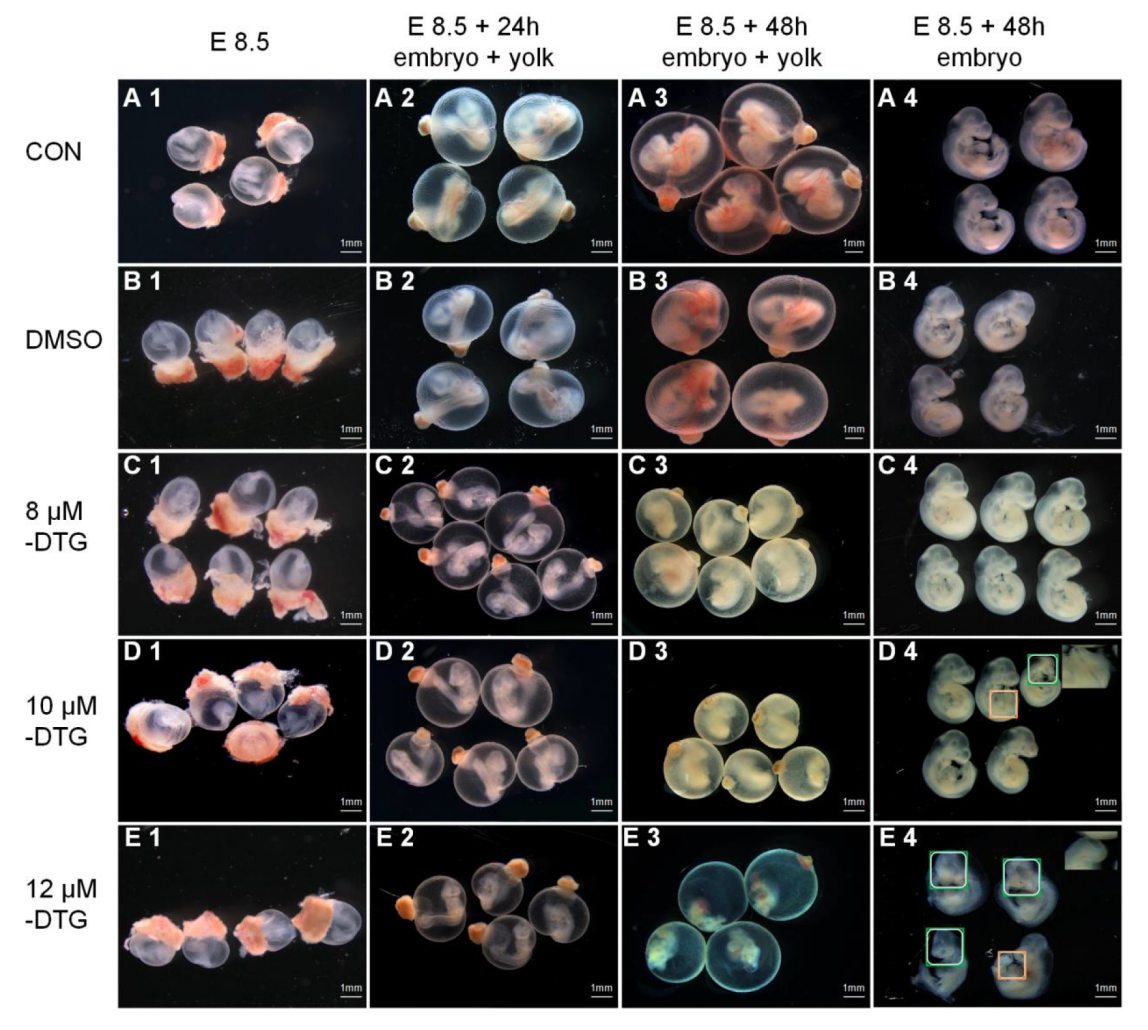
Figure 1 DTG inhibits neural tube development and yolk sac circulation in mouse embryos in a dose-dependent manner via the WEC Mouse embryos were collected at E8.5 (A1–E1), cultured for 24 h (A2–E2) and 48 h with an intact yolk sac (A3–E3), and then, they were imaged after the yolk sac and amnion (A4–E4) were removed. (A–E) Embryos treated with control medium (A), DMSO vehicle (B), or DTG at 8 μM (C), 10 μM (D) or 12 μM (E). Scale bar: 1 mm. In D4 and E4, boxed regions denote cranial and caudal neural tube defects, and righthand insets show magnified views of the unclosed caudal neuropore.

DTG significantly disrupted neural tube development and yolk sac circulation in mouse embryos in a dose-dependent manner. At 10 μM, the incidence of NTDs and circulatory abnormalities increased notably. ( Figure 1). To assess the dose-dependent effect of DTG on embryonic development, we employed a well-established WEC system. Mouse embryos with 4–5 somite pairs were chosen for culture ( Figure 1A1–E1). The DTG concentration range was intended to represent levels below, at, and above the clinical maximum blood concentration, thereby offering a comprehensive evaluation of its embryotoxic potential. After 24 h of culture, yolk sac circulation in control embryos ( Figure 1A,B) was partially established, with the formation of advanced vascular branches. Arterial and venous poles appeared, the heart chambers were filled with blood, and strong heartbeats were observed ( Supplementary Video: animated version of Figure 1A3, E8.5 + 48 h). Initial V-to-C embryonic turning and cranial neural tube closure also occurred ( Figure 1A2,B2). By 48 h, the yolk sac vasculature had developed larger branches, leading to the formation of the yolk stalk ( Figure 1A3,B3). Functional blood circulation was achieved through the heart chamber, umbilical cord, and yolk sac vessels, accompanied by vigorous heartbeats. Closure of neural tubes in the cranial and caudal regions and the appearance of limb buds were evident ( Figure 1A4,B4). In embryos treated with 8 μM DTG, after 24 and 48 h of culture, yolk sac vascular development was impaired, resulting in fewer branches and a pale appearance ( Figure 1C2,C3). Nonetheless, some vascular formation was present, although arterial and venous poles were absent. Morphologically, the embryos appeared normal, with successful cranial and caudal neural tube closure and primary structure formation ( Figure 1C4). In the 10 μM DTG-treated group, the yolk sacs were uniformly pale, indicating poor circulation ( Figure 1D2,D3). Partial embryonic turning occurred, although the turning rate significantly decreased compared with that of the controls. After 48 h, the yolk sac diameter decreased ( Figure 1D3), the developmental scores significantly decreased, and NTDs were evident, with unclosed caudal regions and cranial malformations ( Figure 1D4). The incidence of neural tube defects reached 50% ( Table 1). At 12 μM DTG, yolk sac circulation was severely compromised ( Figure 1E3,E4), and embryonic resorption was observed in some embryos. After 48 h, severe cranial malformations were apparent ( Figure 1E4), with increased numbers of unclosed caudal neural tubes. The neural tube defect rate increased to 85% ( Table 1).

**Table:** **Table 1** DTG impairs neural tube closure in embryos by the whole embryo culture

Dose	Litters ( *n*)	Number of embryos cultured	Caudal neural tube defects ( *n*)	Brain neural tube defects ( *n*)	NTDs (%)	Abnormal YS circulation (%)
Control	3	16	0	0	0	0
DMSO (1.1 mg/mL)	3	16	0	0	0	0
8 μM DTG	4	20	0	0	0	4*
10 μM DTG	3	18	2	7	50**	17**
12 μM DTG	4	20	4	13	85**	20***

Two datasets were analyzed by Student’s *t* test. Quantitative data are expressed as the mean ± SEM. * *P*  < 0.05, ** *P*  < 0.01, *** *P*  < 0.001.

### Dose-dependent toxicity and NTDs induced by intraperitoneal injection of DTG in mice

**Figure 2:**
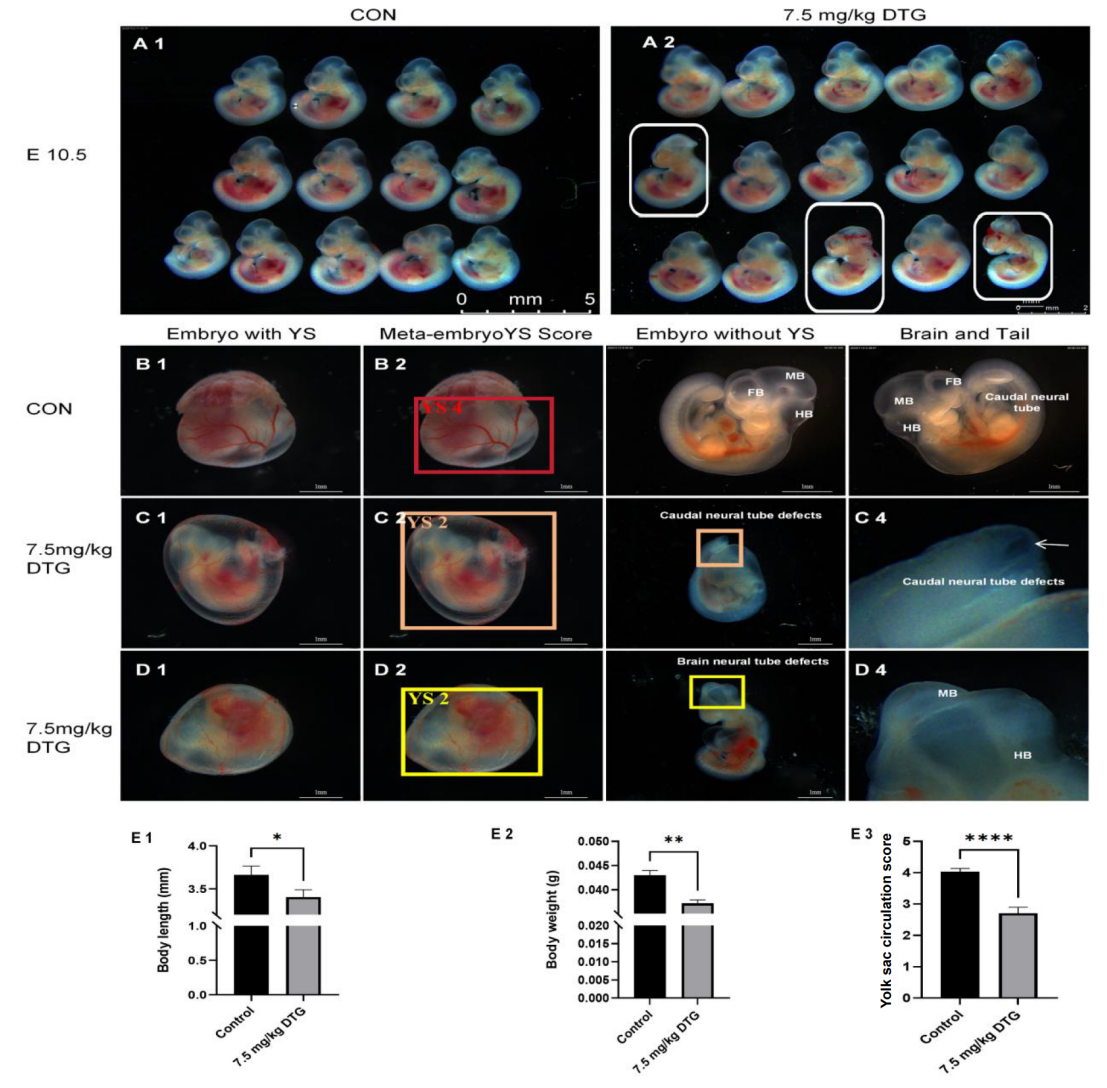
Figure 2 Dose-dependent toxicity and NTDs induced by intraperitoneal injection of DTG in mice Intraperitoneal DTG (7.5 mg/kg) induced neural tube and yolksac defects in mouse. Embryos mouse dams received an intraperitoneal injection of vehicle (control) or DTG (7.5 mg/kg and 15 mg/kg) at E 8.5. All the litters were harvested 48 h later. (A1,A2) Wholelitter overview of embryos with the yolk sac and amnion removed. The white boxes in A2 highlight cranial NTDs in DTG-treated embryos. (B1–D1) Control (B1) and DTG-treated (C1,D1) embryos retained an intact yolk sac. (B2–D2) Yolksac (YS) development scores (1–5 scale) in the control (B2) and DTG groups (C2,D2). (B3–D3) Embryos after removal of the yolk sac and amnion, showing normal morphology in the control group (B3) and NTDs in the DTG groups (C3,D3). Boxes in C3 and D3 denote caudal and cranial neuropore defects, respectively. (C4,D4) Highmagnification insets of boxed NTD regions from (C3,D3). Scale bar: 1 mm. (E1–E3) Quantify the embryo crown-rump length (E1), wet weight (E2), and YS score (E3). Embryo length, weight, and YS score in DTG-treated embryos were significantly reduced versus control (length and weight date are presented as the mean ± SEM. *P < 0.05, **P < 0.01, unpaired twotail Student's t test; yolk sac score used the nonparametric Mann-Whitney U test, ****P < 0.0001).

Intraperitoneal injection of DTG at both low and high clinical doses had marked embryotoxic effects on E10.5 mouse embryos. DTG dose-dependently increased NTD incidence, inhibited growth, induced yolk sac circulation abnormalities, and impaired overall embryonic development. In the control group ( Figure 2A1), no NTDs were noted, and yolk sac circulation was fully vascularized, displaying multiple branching levels. Large blood vessels formed with clearly defined arterial and venous poles ( Figure 2B1,B2). Embryonic brain vesicles developed well, and tailbud closure was complete ( Figure 2B3,B4). In the low-clinical-dose group (7.5 mg/kg DTG), continuous intraperitoneal injection for 3 days caused neural tube closure defects ( Figure 2A2). The yolk sac vasculature was patchy and uneven, with thin, poorly branched vessels ( Figure 2C1,D1). Embryos presented incomplete cranial ( Figure 2D3) and caudal neural tube closure ( Figure 2C3), reduced body length ( Figure 2E1), decreased weight ( Figure 2E2), and lower yolk sac scores ( Figure 2E3). Yolk sac circulation scores decreased from 4 points in the control group to approximately 2 points ( Figure 2C2,D2). In the high-clinical-dose group (15 mg/kg DTG), the abnormalities were more severe than they were in the low-dose group ( Table 2). The embryo resorption rates increased, whereas the incidence of neural tube defects decreased slightly. Yolk sac circulation abnormalities were similar between the low- and high-dose groups (9.52% vs 10.26%). These findings indicate that DTG significantly affects embryonic development in a dose-dependent manner, affecting neural tube closure, yolk sac circulation, and overall growth.

**Table:** **Table 2** Toxicity of DTG on neural tube closure at E10.5

Dose	Number of pregnant dams	Number of embryos	Normal (%)	NTDs (%)	Absorption (%)	Abnormal yolk sac circulation
Control	4	44	44 (100.00)	0	0	0
DMSO (0.55 g/kg)	4	43	43 (100.00)	0	0	0
DTG (7.5 mg/kg)	4	42	37 (88.10)	4 (9.52)*	1 (2.38)	4 (9.52)*
DTG (15 mg/kg)	4	39	30 (76.92)	7 (17.95)**	2 (5.13)	4 (10.26)*

Two datasets were analyzed by Student’s *t* test. Quantitative data are expressed as the mean ± SEM. * *P*  < 0.05, ** *P*  < 0.01.

### DTG increases the ROS levels in embryos

**Figure 3:**
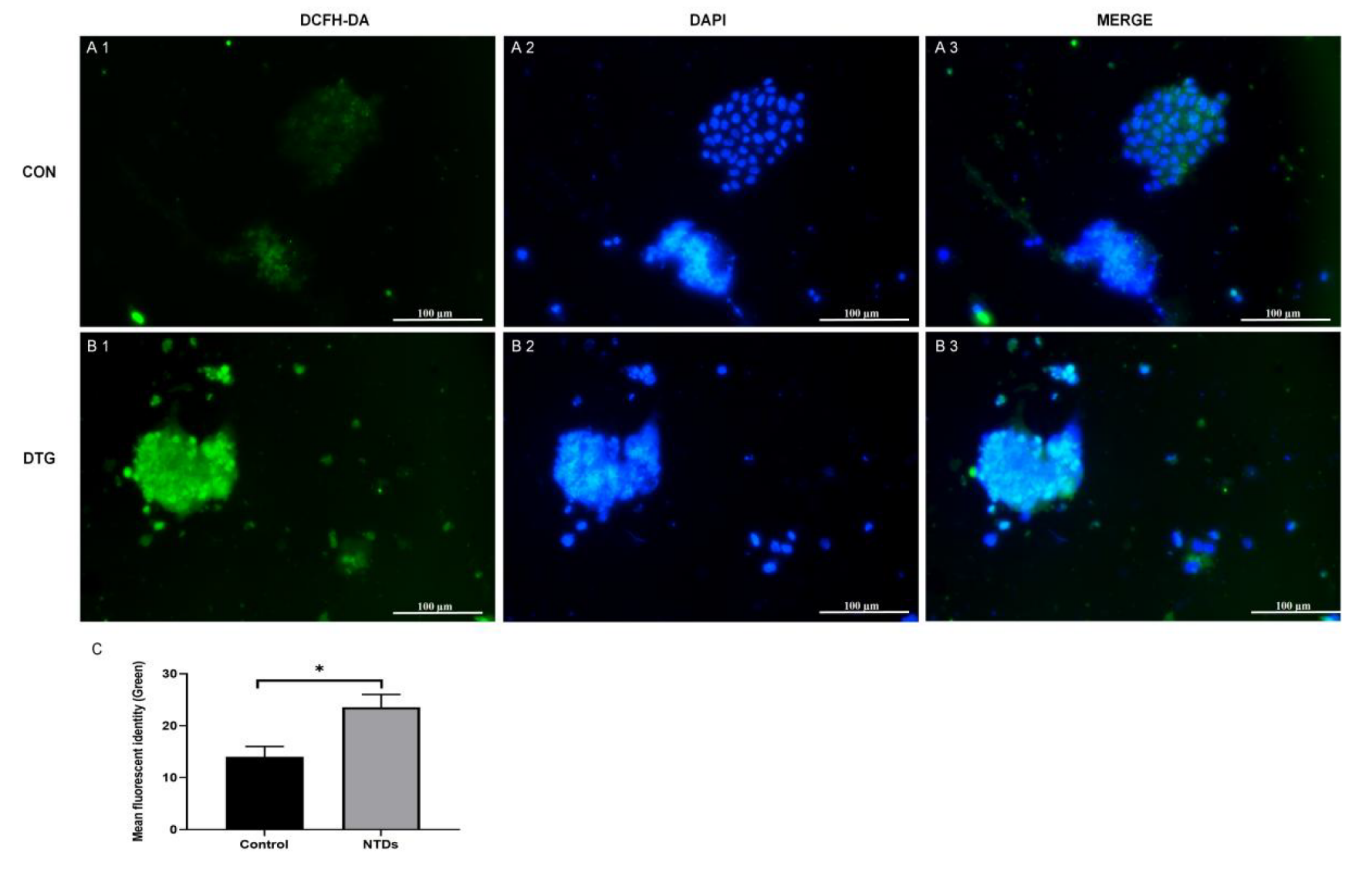
Figure 3 DTG exposure increases ROS levels (A1,B1) Representative images of DCFH-DA staining of E10.5 neuroepithelial cells showing green fluorescence. (A2,B2) All the cell nuclei were stained with DAPI (blue). (A3,B3) Merged images of green fluorescence (DCFH-DA) and blue fluorescence (DAPI). Scale bar: 100 μm. (C) Quantification of green fluorescence signals overlapping with blue nuclei. *P < 0.05 indicates a significant difference compared with the control groups.

To assess oxidative stress, DCFH-DA staining was performed on E10.5 mouse neuroepithelial cells. As shown in Figure 3, compared with control embryos, DTG-treated embryos presented stronger green fluorescence signals ( Figure 3A1,B1), indicating increased ROS production. Nuclear staining with DAPI ( Figure 3A2,B2) confirmed the cellular localization of the protein. The merged images ( Figure 3A3,B3) clearly overlap the green and blue signals. Quantitative analysis revealed a significant increase in green fluorescence intensity overlapping with DAPI-stained nuclei in the DTG group, suggesting increased intracellular ROS generation ( Figure 3C; *P*  < 0.05 vs control).

### DTG activates the Nrf2 pathway with imbalanced antioxidant enzyme expression in NTD embryos

**Figure 4:**
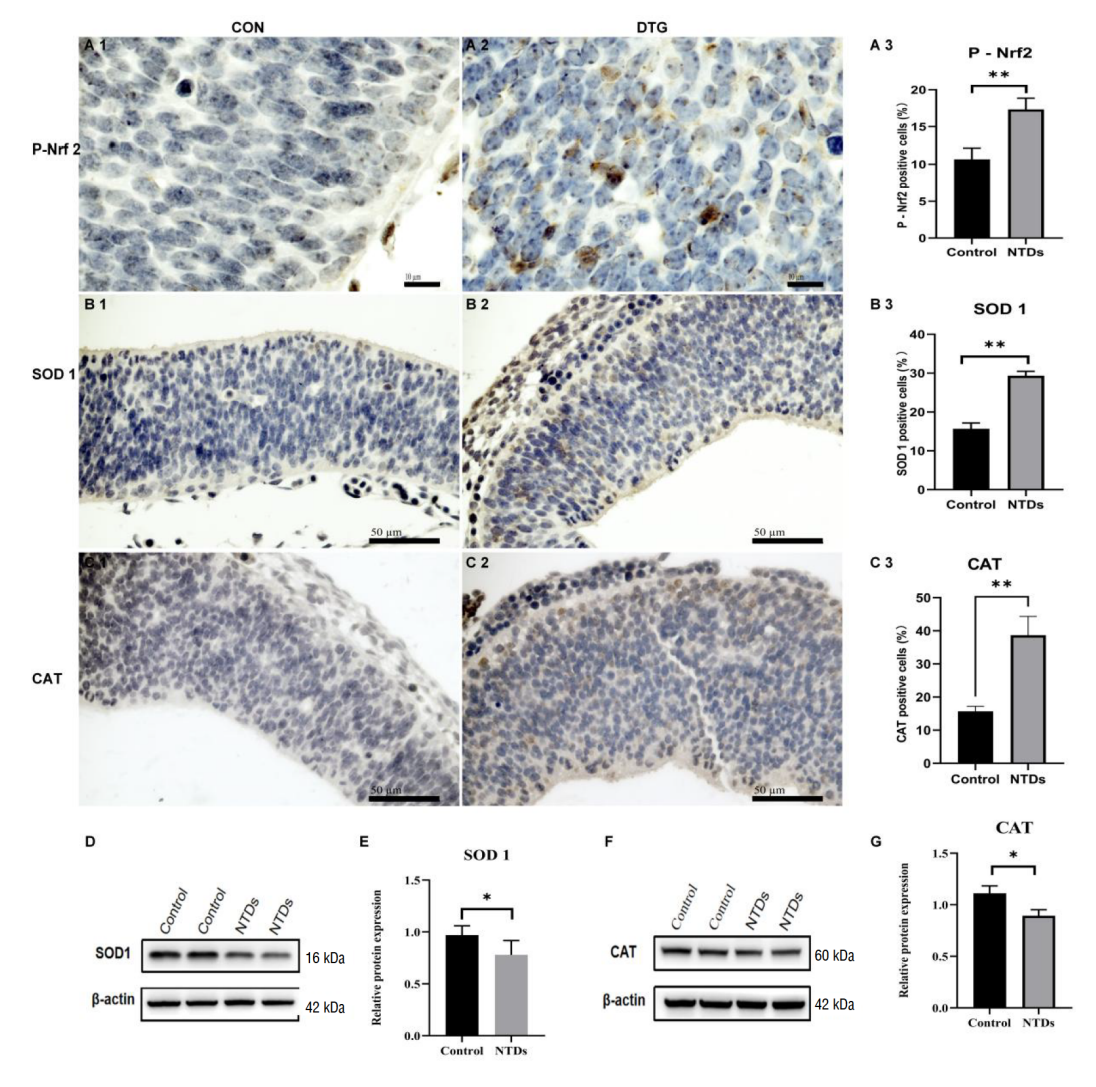
Figure 4 DTG activates the Nrf2 pathway with imbalanced antioxidant enzymes SOD1 and CAT in the mouse embryonic brain (A) Immunohistochemical staining of phosphorylated Nrf2 (P-Nrf2) in control (A1) and DTG-treated (A2) embryos. (A3) Quantification of P-Nrf2-positive cells. Data are presented as the mean ± SEM and were compared by unpaired twotailed Student’s t test. *P < 0.05. Scale bar: 10 μm. (B,C) Immunohistochemical staining for SOD1 (B1, control; B2, NTDs) and CAT (C1, control; C2, NTDs) in neural tube sections at E11.5. Scale bar: 50 μm. Nrf2+ (A3), SOD1+ (B3) and CAT+ (C3) cells were quantified as a percentage of total nuclei in control versus NTD tissues. Data are presented as the mean ± SEM; **P < 0.01 according to an unpaired twotailed Student’s t test. (D,E) Western blot analysis of SOD1 and β-actin expression levels in control and NTD tissues. SOD1 protein levels were normalized to those of β-actin. (F,G) Western blot analysis of CAT and β-actin levels. CAT levels were normalized to those of β-actin. Data are presented as the mean ± SEM; *P < 0.05 according to the unpaired twotailed Student’s t test.

DTG treatment markedly activated the Nrf2 pathway. Immunohistochemical analysis showed a significant increase in the proportion of P-Nrf2–positive cells in DTG-treated embryos compared with controls ( Figure 4A1–A3). Consistent with this activation, qPCR revealed significantly elevated mRNA levels of Sod1, Cat, and Gpx3 in the NTD group ( Supplementary Figure S1). Correspondingly, immunohistochemical staining demonstrated enhanced SOD1 ( Figure 4B1–B3) and CAT ( Figure 4C1–C3) signals in the neuroepithelium of NTD embryos, with significantly higher proportions of positive cells (P < 0.01). In contrast, Western blot analysis showed that the total protein levels of SOD1 ( Figure 4D,E) and CAT ( Figure 4F,G) were reduced in NTD tissues. These data indicate Nrf2 activation accompanied by imbalanced expression of antioxidant enzyme in NTD embryos ( Figure 4).

### DTG impairs neural tube development in E11.5 mouse embryos by inhibiting proliferation and promoting apoptosis

**Figure 5:**
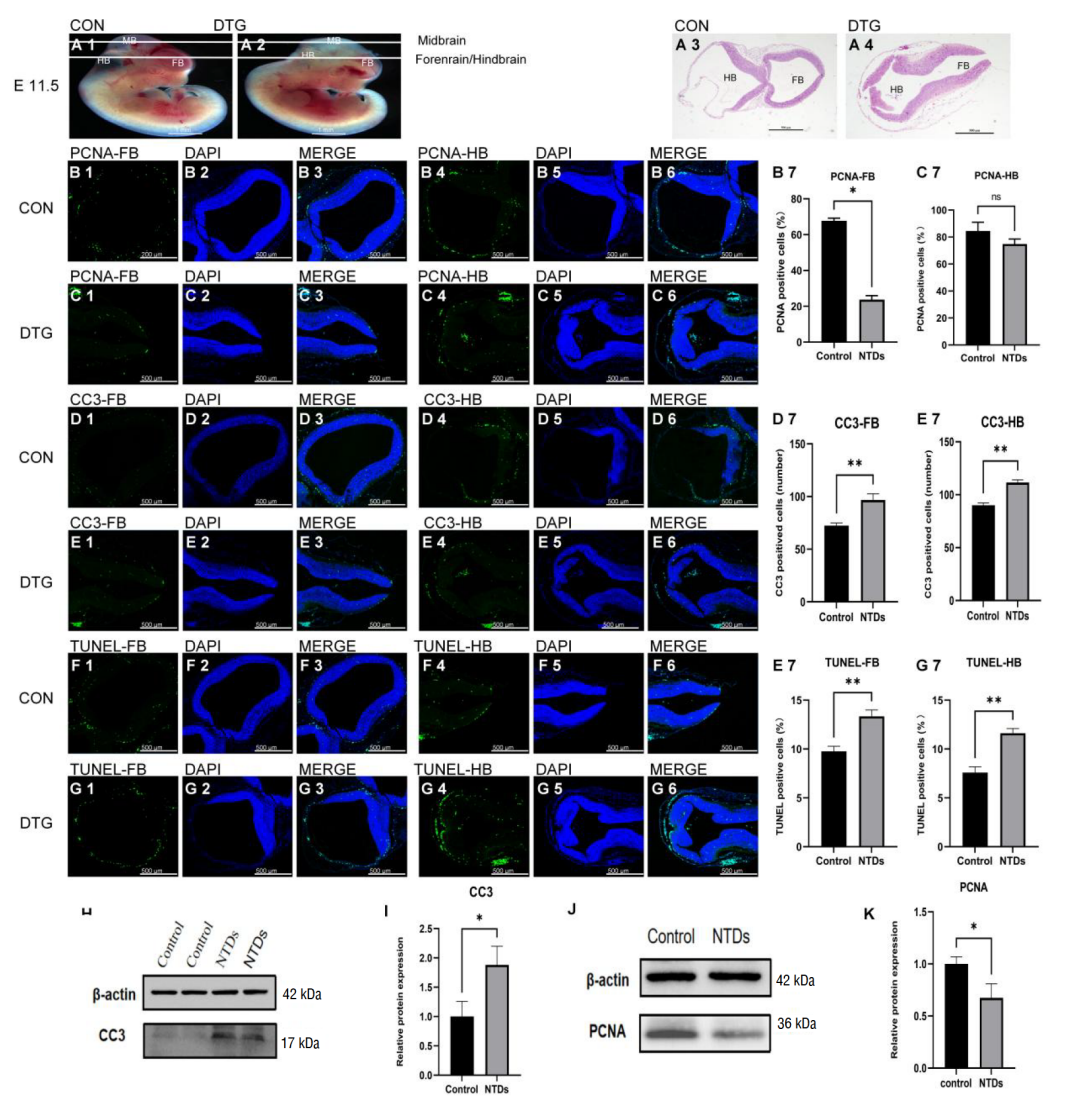
Figure 5 DTG impairs neural tube development in E11.5 mouse embryos by inhibiting proliferation and promoting apoptosis (A1,A2). Brightfield images of control (A1) and DTG-treated (7.5 mg/kg; A2) E11.5 embryos after yolksac and amnion removal; arrows indicate forebrain (FB) and hindbrain (HB) regions. (A3–A4) HEstained coronal sections through FBs and HBs in control (A3) and DTG (A4) embryos. Scale bar: 500 μm. (B1–C6) Immunofluorescence staining for the proliferation marker PCNA (green) and DAPI (blue) in FBs (B1–B3, C1–C3) and HBs (B4–B6, C4–C6) from control (B) and DTG (C) embryos. Scale bar: 200 μm. (B7–C7) Quantification of PCNA+ cells (% of DAPI+ nuclei) in FBs and HBs. *P < 0.05; ns, not significant. (D1–E6) CC3 (green) and DAPI (blue) staining of FBs (D1–D3, E1–E3) and HBs (D4–D6, E4–E6) from control (D) and DTG (E) embryos. Scale bar: 200 μm. (D7–E7) Quantification of CC3+ cells (number per field) in FBs and HBs. **P < 0.01. (F1–G6) TUNEL assay (green) and DAPI (blue) in FBs (F1–F3, G1–G3) and HBs (F4–F6, G4–G6). Scale bar: 200 μm. (F7–G7) Quantification of TUNEL+ cells (% of DAPI+ nuclei) in FBs and HBs. **P < 0.01. (H,I) Western blot analysis of CC3 and βactin (H), with densitometric analysis (I) normalized to β-actin. *P < 0.05. (J,K) Western blot analysis of PCNA and βactin (J), with quantification (K). *P < 0.05.

To investigate the effects of DTG on embryonic development, particularly neural tube closure, we administered low clinical doses (7.5 mg/kg) of DTG and collected embryos at E11.5. In the control group, the embryos displayed well-formed neural tubes with distinct segmentation into the forebrain, midbrain, and hindbrain ( Figure 5A1). The forebrain prominently extended into a horn-like structure resembling the telencephalon, whereas the midbrain and hindbrain were well defined, with a thin, translucent roof over the hindbrain. Conversely, embryos subjected to DTG presented underdeveloped forebrains and flattened hindbrains ( Figure 5A2). Paraffin section analysis revealed that control embryos developed clear demarcations between forebrain and hindbrain vesicles, with the latter being thin-walled, rounded, and hollow ( Figure 5A3). In contrast, DTG-treated embryos presented poorly defined forebrain and hindbrain vesicles, with variably shaped hindbrain lumens ( Figure 5A4).

Immunofluorescence analysis of neural tube epithelial cells revealed significant changes in proliferation and apoptosis rates. Compared with control embryos ( Figure 5B1–B6, D1–D6, and F1–F6), DTG-treated embryos presented a significant increase in apoptosis, as detected by cleaved caspase-3 ( Figure 5D7,E1–E7) and TUNEL assays ( Figure 5E7,G1–G7), across both forebrain and hindbrain regions. Proliferation, as assessed by immunostaining, was significantly reduced in the forebrain ( Figure 5B7,C1–C3) and non-significantly decreased in the hindbran ( Figure 5C4–C7). Western blot analysis further confirmed increased CC3 ( Figure 5H,I) and decreased PCNA ( Figure 5J,K) expression levels in the NTD group. As shown in Table 3, the incidence of cranial NTDs was 20.5%, the abnormal yolk sac circulation rate was 11.76%, and the embryo resorption rate was 2.94%. These results illustrated that DTG affects neural tube development, primarily through reducing cell proliferation and enhancing apoptosis in neural tube epithelial cells, with marked effects in the forebrain and hindbrain regions.

**Table:** **Table 3** Toxicity of DTG on neural tube closure at E11.5

Dose	Number of pregnant dams	Number of embryos	Normal (%)	NTDs (%)	Absorption (%)	Abnormal yolk sac circulation (%)
Control	3	37	37 (100.00)	0	0	0
DTG 7.5 (mg/kg)	3	34	26 (76.47)	7 (20.5)**	1 (2.94)	4 (11.76)*

Two datasets were analyzed by Student’s *t* test. Quantitative data are expressed as the mean ± SEM. * *P*  < 0.05, ** *P*  < 0.01.

### DTG induces structural disruption, decreases proliferation and angiogenesis, and increases apoptosis in the yolk sac

**Figure 6:**
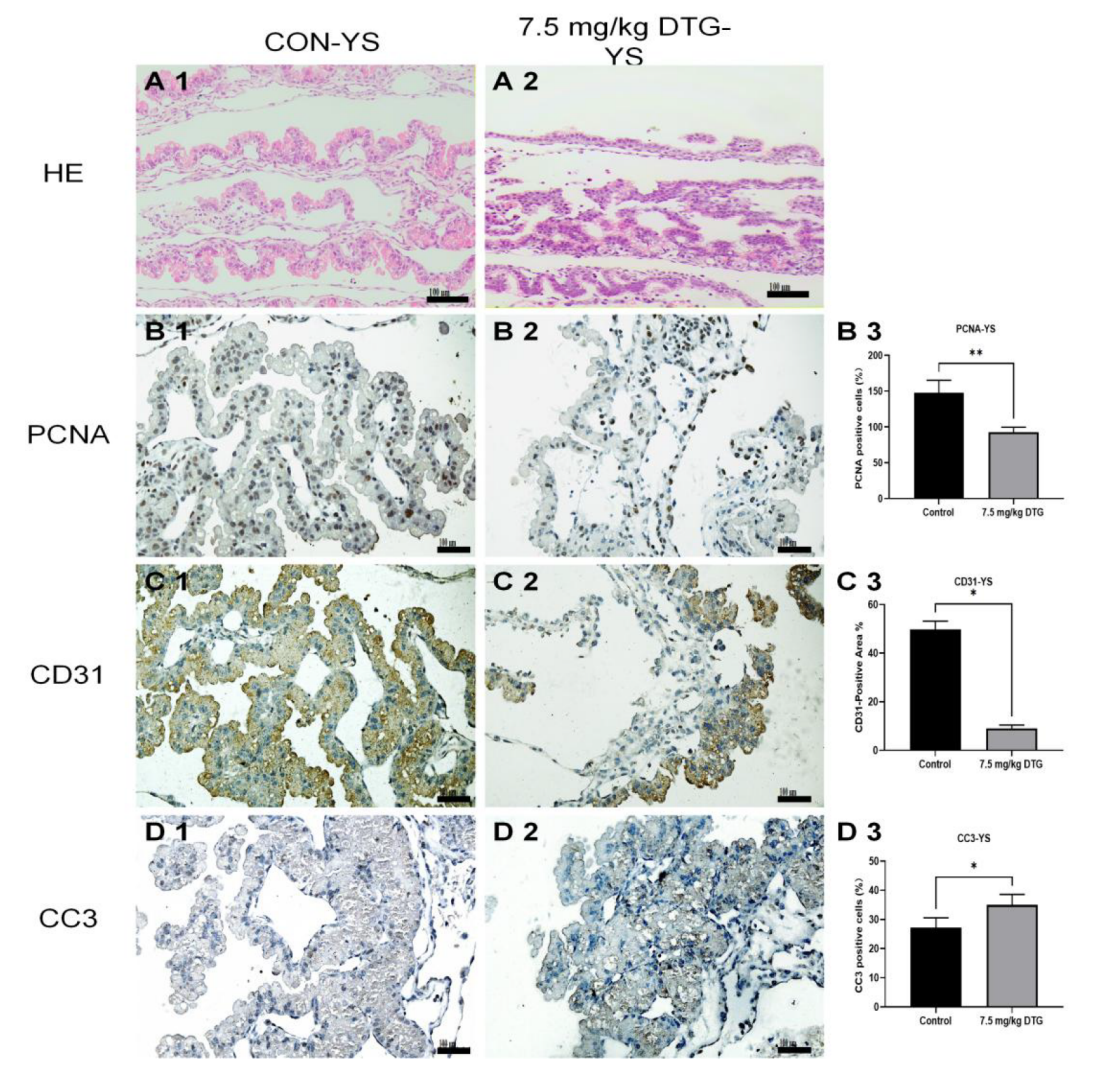
Figure 6 DTG induces structural disruption, decreases proliferation and angiogenesis, and increases apoptosis in the yolk sac (A) HE staining revealed structural differences in yolk sacs between the control group (A1) and the DTG-treated group (A2). (B) PCNA staining indicated a reduction in cell proliferation in the DTG group (B2), with a significant decrease in PCNA expression (B3, **P < 0.01). (C) CD31 staining demonstrated reduced vascularization in the DTG-treated group (C2), with fewer CD31-positive cells (C3, *P < 0.05). (D) CC3 staining revealed increased apoptosis in the DTG group (D2), along with a significant increase in CC3-positive cells (D3, *P < 0.05). Scale bar: 100 μm.

The administration of 7.5 mg/kg DTG led to the disruption of yolk sac morphology and function, likely by inhibiting cellular proliferation, impairing angiogenesis, and increasing apoptosis in E11.5 embryos. Histological examination with HE staining revealed significant structural differences between the control ( Figure 6A1) and DTG-treated groups ( Figure 6A2). In the control group, the columnar epithelial cells of the yolk sac were orderly, forming a “wave-like” loose structure akin to a “wreath”. The mesothelial cells beneath were loosely connected to the connective tissue, with prominent large blood vessels containing flowing red blood cells. In contrast, the DTG-treated group presented a denser tissue structure with disorganized columnar epithelial arrangements, tightly adhered mesothelial and connective tissues, and a lack of large blood vessel formation, resulting in compact “cord-like” structures ( Figure 6A2). Immunohistochemical staining for PCNA revealed a significant decrease in the number of PCNA-positive cells in the DTG-treated group, indicating reduced cell proliferation ( Figure 6B1–B3; *P*  < 0.01). Additionally, CD31 immunostaining revealed a significantly reduced CD31-positive area, indicating impaired angiogenesis ( Figure 6C1–C3; *P*  < 0.05). CC3 staining revealed a substantial increase in CC3-positive cells in the DTG group compared with the control group, indicating increased levels of apoptosis ( Figure 6D1–D3; *P*  < 0.05). These findings suggested that DTG compromised yolk sac integrity and function by impairing cellular proliferation, inhibiting angiogenesis, and promoting apoptosis, ultimately affecting embryonic development.

## Discussion

In this study we examined the effects of DTG on embryonic neural tube development in mice using both *in vitro* and *in vivo* models to establish a reliable model for NTDs. We found that DTG at concentrations of 10 μM or higher significantly induced NTDs. However, *in vivo* experiments suggested that maternal metabolism significantly reduced embryonic exposure levels, which mitigated potential risks. DTG appeared to induce NTDs by increasing oxidative stress and enhancing neural tube epithelial apoptosis, offering key insights into the safety of DTG during pregnancy.

NTDs, including spina bifida, cranial bifida, and others, are the second most common congenital malformations [ 1– 3] . WEC technology is pivotal for targeting the critical window of neural tube closure, providing a robust platform to study NTD pathogenesis and safety thresholds for therapeutic drugs [ 17, 31– 33] . In this study, exposure to 8 μM DTG for 48 h in WECs did not result in abnormal neural tube development compared with that in the control group. The embryos consistently presented E10.5 developmental characteristics, including prosencephalon formation, vesicular mesencephalon, complete somite elongation, axial rotation, and fully closed tail tubes. However, the scores for yolk sac vascular circulation were significantly reduced, indicating that even low concentrations of DTG might have affected vascular development. At a DTG concentration of 10 μM, although axial rotation was complete, NTDs such as cerebellar abnormalities and unclosed tail tubes were observed, indicating that this concentration exceeded the safety threshold. LaNoce *et al*. [35] reported that treatment with 10 μM DTG significantly reduces the volume of forebrain organoids, decreases the number of neural tube-like structures, and downregulates the expression of neurogenesis-related genes. These findings were consistent with our results. Posobiec *et al*. [18] reported that culturing rat embryos with DTG at a concentration of 9.3 μg/mL did not result in developmental toxicity. Daniela *et al*. [36] demonstrated that DTG, even at a low dose (1 μM), could impair neural system development in zebrafish embryos. These differences might be resulted from interspecies variation and the timing of drug exposure. However, it is important to note that the direct exposure method used in this study did not consider maternal metabolism, which limits the clinical relevance of the results ( Supplementary Table S2).

To address this limitation, we conducted *in vivo* experiments to evaluate the embryotoxicity of DTG within the context of maternal metabolism. An intraperitoneal injection of 7.5 mg/kg DTG, a dose approximating clinical levels, resulted in a 9.52% rate of NTDs, which was significantly lower than the 50% observed with 10 μM DTG *in vitro*. These findings underscore the protective role of maternal metabolism. Although the developmental processes in mice are similar to those in humans [19], the observed NTD rates are higher than those reported in the Botswana study (0.19%–0.3%) [ 10, 11] . This difference was likely due to metabolic transport [13]. Mohan *et al*. [37] reported that pregnant mice administered with DTG at a dose of 2.5 mg/kg, equivalent to therapeutic levels in humans, exhibited a marked increase in the incidence of NTD. Higher-than-clinical doses of DTG did not significantly increase the incidence of NTDs but did not result in an increased resorption rate, which was consistent with our findings [ 11, 38] ( Supplementary Tables S3 and S4).

Our results showed that DTG exposure increased ROS levels in E10.5 mouse embryos, indicating enhanced oxidative stress during early development. Excessive ROS have been linked to neural tube defects and impaired embryogenesis [39]. These findings suggest that oxidative stress could be a key mechanism in DTG-related teratogenicity. We found that DTG exposure activated the Nrf2 pathway, accompanied by imbalanced expressions of downstream antioxidant enzymes such as SOD1 and CAT. Nrf2 is a central regulator of antioxidant defenses [22], but its insufficient or dysregulated activation has also been associated with developmental toxicity [22]. In our experiments, DTG-treated embryos still exhibited reduced cell proliferation and increased apoptosis. These findings are consistent with previous studies showing that oxidative stress-induced apoptosis impairs the cellular structure necessary for proper neural tube closure [ 20, 21] and further support the conclusion that Nrf2 activation alone is inadequate to counteract DTG-induced oxidative stress during embryogenesis [ 22, 40] . Taken together, these findings underscore the importance of enhancing embryonic antioxidant capacity during DTG-based antiretroviral therapy. Folic acid, which has been widely recommended for NTD prevention, also exhibits antioxidant activity and might offer additional protection against DTG-induced developmental toxicity [41]. Future studies involving folic acid rescue or Pax3 overexpression will be critical to confirm the mechanistic role of DTG-induced oxidative stress and Pax3 downregulation in NTD pathogenesis.

Our experimental results revealed that the yolk sac vasculature was damaged, the yolk sac structure was disrupted, and the proliferation-apoptosis balance was disrupted following DTG treatment. The yolk sac, one of the earliest structures to form during development [42], plays a critical role in providing essential nutrients and serves as the primary placenta for gas exchange and nutrient uptake [ 42, 43] . Its blood vessels are crucial for early circulation and organ formation [ 30, 43] , and any dysfunction can lead to malformations or developmental delays [30]. Teratogens that disrupt yolk sac function may result in fetal abnormalities, highlighting the significance of the yolk sac in proper development [ 34, 44] . Our *in vivo* and *in vitro* experiments substantiated that impairments in yolk sac circulation are likely a primary cause of abnormal embryonic development. Mohan *et al*. [37] reported that the DTG-treated group exhibited vascular abnormalities, mirroring our findings and supporting the embryonic vascular toxicity of DTG. The yolk sac is involved in nutrient transport, and hematopoietic system derivation vascular developmental abnormalities can also lead to embryonic developmental defects [ 30, 45] . Future work will include functional validation to confirm yolk sac vascular defects.

In summary, this study provides valuable insights into the developmental toxicity of DTG by integrating *in vitro* and *in vivo* approaches. This study established a safe concentration and underscores the need for further research into DTG-induced NTD mechanisms. Future studies should also assess the impact of maternal metabolism on drug distribution to optimize pregnancy-related drug regimens.

## Supporting information

25213Supplementary_CONTROL

25213_Supplementary_Tables(1)

## Supplementary Data

Supplementary data is available at *Acta Biochimica et Biophysica Sinica* online.
