# Supplementary material for: Combined ex vivo and in vivo evaluation of dolutegravir embryotoxicity: NTDs and yolk sac vascular abnormalities : Dolutegravir induces NTDs and YS abnormalities
Source: Acta Biochim Biophys Sin (Shanghai). 2025 Nov 3;58(3):530–40. doi: 10.3724/abbs.2025142 (PMC13059749; doi:10.3724/abbs.2025142)
Supplement: 25213_Supplementary_Tables(1) [file 25213_Supplementary_Tables(1).docx]

**Supplementary Table S1. Sequences of primers used in this study**

| Gene | Primer sequence (5′→3′) |
| --- | --- |
| *β-actin* | GATTACTGCTCTGGCTCCTA ATCGTACTCCTGCTTGCTGA |
| *Gpx1* | TCTCTGAGGCACCACGATCCG |
|  | ACCGAGCACCACCAGTCCAC |
| *Gpx3* | TGACATCCGCTGGAACTTTGAGAAG |
|  | CTGCCTGCCGCCTCATGTAAG |
| *Sod1* | AAGCGGTGTGCGTGCTGAAG |
|  | TGTCCTGACAACACAACTGGTTCAC |
| *Sod2* | TCCCAGACCTGCCTTACGACTATG |
|  | CTCCTCGGTGGCGTTGAGATTG |
| *Cat* | GGCGGGAACCCAATAGGAGATAAAC |
|  | TGTCAAAGTGTGCCATCTCGTCAG |
| *Pax3* | CAGTGAGTTCTATCAGCCGCATCC |
|  | TTCTCGCTTTCTTCTGCTTCCTTCC |

| **Supplementary** **Table S2. Plasma and tissue distribution of DTG in maternal, fetal, and infant** | | | | |
| --- | --- | --- | --- | --- |
| Sample type | DTG concentration | Range | Remarks/ratio | Reference title |
| Non-pregnant adult plasma | Cmax ≈ 6.16 μg/mL Ctrough ≈ 1.64 μg/mL | – | Steady-state after 50 mg daily oral dosing | [Safety, tolerability, and pharmacokinetics of dolutegravir in treatment-naive adults with HIV](https://www.ncbi.nlm.nih.gov/pmc/articles/PMC3805712) |
| Pregnant plasma | Cmax ≈ 3.15 μg/mL Ctrough ≈ 0.68 μg/mL | – | AUC reduced to ~53% of non-pregnant value | Pharmacokinetics of Dolutegravir in Pregnant Women |
| Cord blood | Median: 1.67 μg/mL | 1.17–2.00 | Cord/maternal ratio ≈ 1.25 | Dolutegravir pharmacokinetics in pregnant and postpartum women living with HIV. |
| Maternal plasma (at delivery) | Median: 1.24 μg/mL | 0.57–1.68 μg/mL | – | Same as above |
| neonatal plasma (post-birth) | Cmax ≈ 1.85 μg/mL | – | Half-life ≈ 32.8 hours | Same as above |
| Breast milk | – | – | Milk-to-plasma ratio ≈ 0.03 | Perinatal antiretroviral guidelines – Dolutegravir (US NIH Clinicalinfo, 2024) |
| Infant plasma (via breastfeeding) | Median: 66.7 ng/mL | 21–654 ng/mL | – | Same as above |

| **Supplementary** **Table S3. Overview of NTD risk linked to DTG exposure in clinical and animal studies**  **(No observable effect on embryonic development)** | | | | | | |
| --- | --- | --- | --- | --- | --- | --- |
| Study type | DTG dose | Exposure timing | NTD incidence | Notes | Title | Time |
| Botswana Tsepamo | Standard human dose (50 mg/day) | Approximately 28 days after conception | Initial report: 0.94% ,Updated: 0.11% | Early data suggested an increased risk, but subsequent larger datasets showed no significant difference | Neural-Tube Defects with Dolutegravir Treatment from the Time of Conception | 2018 Jul 24 |
| U.S. CDC Surveillance | Standard human dose (50 mg/day) | At conception | 7.0 per 10,000 live births | [NTD prevalence among HIV-exposed pregnancies is similar to that in the general population..](https://www.cdc.gov/mmwr/volumes/69/wr/mm6901a1.htm) | Neural Tube Defects in Pregnancies Among Women With Diagnosed HIV Infection — 15 Jurisdictions, 2013–2017 | 2020 Jan 1 |
| Brazilian National Cohort | Standard human dose (50 mg/day) | Within 8 weeks of estimated date of conception | 0.18% (2/1117) | No significant increase in NTDs was observed in a population with folic acid fortification. | Dolutegravir and pregnancy outcomes in women on antiretroviral therapy in Brazil: a retrospective national cohort study | 2021 Jan 1 |
| U.S. Medicaid Cohort | Standard human dose (50mg/day) | Periconceptional dolutegravir exposure | 1/993 (~0.10%) | There was no significant difference compared to HIV-negative controls. | Dolutegravir and pregnancy outcomes including neural tube defects in the USA during 2008-20: a national cohort study | 2023 Jul 25 |
| Eswatini Hospital Surveillance | Standard human dose (50 mg/day) | At conception | 0.08% (4/4,902) | NTD rates were similar among deliveries regardless of maternal HIV status and DTG exposure. | Strengthening the Evidence: Similar Rates of Neural Tube Defects Among Deliveries Regardless of Maternal HIV Status and Dolutegravir Exposure in Hospital Birth Surveillance in Eswatini | 2023 Sep |
| C57BL/6J (mouse) | 2.5 -12.5 mg/kg oral gavage | Embryonic days 0.5-15.5 | 0.47% | The NTD rate is similar to that reported in the Tsepamo study. DTG is unlikely to be an inhibitor of folate uptake. | Dolutegravir in pregnant mice is associated with increased rates of fetal defects at therapeutic but not at supratherapeutic levels | 2021 Jan 1 |
| Rat WEC | 5.3-9.3 μg/mL | Embryonic days 9-11 | No observable developmental toxicity | The exposure levels observed were comparable to those in clinical scenarios. | No developmental toxicity observed with dolutegravir in rat whole embryo culture | 2021 Aug 28 |

| **Supplementary** **Table S4. Overview of NTD risk linked to DTG exposure in clinical and animal studies**  **(Evidence of neural tube developmental abnormalities)** | | | | | | |
| --- | --- | --- | --- | --- | --- | --- |
| Study type | DTG dose | Exposure timing | NTD incidence | Notes | Title | Time |
| Case report | Clinical | Periconception | 2 cases reported | Suggests potential association between DTG and NTDs | Two cases of neural tube defects with dolutegravir use at conception in south Brazil | 2021 |
| In vitro and animal study | Clinical equivalent | Pregnancy | N/A | DTG interacts with placental folate transporters, potentially affecting folate delivery | Interaction between dolutegravir and folate transporters and receptor in human and rodent placenta | 2021 |
| Review | N/A | N/A | Increased | Discusses potential neurodevelopmental effects of INSTIs | HIV-1 Integrase Strand Transfer Inhibitors and Neurodevelopment | 2022 |
| Animal study (zebrafish) | 1 μM | 4–144 hpf | NTDs | Folic acid reverses DTG-induced neurodefects | Dolutegravir and Folic Acid Interaction during Neural System Development in Zebrafish Embryos | 2023 |
| LM/Bc and SWV (mouse) | 750 mg/kg/day | Embryonic days 0.5-9.5 | Higher in magnesium-deficient group | Hypomagnesemia prior to conception increases the risk for NTDs in DTG-exposed mice. | Gene-nutrient interactions that impact magnesium homeostasis increase risk for neural tube defects in mice exposed to dolutegravir (Magnesium-deficient vs. sufficient diets) | 2023 Jun 12 |
| C57BL/6 (mouse) | 2.5-12.5 mg/kg/day (1-5 HED) | Embryonic days 0.5-10.5 | Increased in low folate group, 1.0%-1.3% | Maternal folate deficiency increases the risk of DTG-associated fetal defects. | Folate deficiency increases the incidence of dolutegravir-associated foetal defects in a mouse pregnancy mode l(Low folic acid (0.3 mg/kg) vs. normal (3 mg/kg)) | 2023 Aug 14 |
| CD-1 (mouse) | Human-equivalent and supratherapeutic doses | Embryonic days 6.5-12.5 | Exencephaly observed in low folate group | Adequate dietary folic acid during pregnancy mitigates developmental defects from DTG exposure. | Dolutegravir-induced neural tube defects in mice are folate responsive | 2024 Feb 29 |
| Organoid | 10 μM | Early neurogenesis stage | Reduced organoid volume | fewer neural rosettes, downregulated neurogenesis genes | Exposure to the antiretroviral drug dolutegravir impairs structure and neurogenesis in a forebrain organoid model of human embryonic cortical development | 2024 |
| In vitro study (organoids) | 10 μM | Early brain organoid development stage | DTG disrupts neurogenesis-related genes | DTG upregulates FOLR1 and alters neurogenesis gene expression | Dolutegravir induces FOLR1 expression during brain organoid development | 2024 |

**Supplementary** **VEDIO** In the 48-h video of the control group embryos, we clearly observed embryonic growth, including successful rotation, development of various organs, and well-defined yolk sac vasculature with visible blood flow. The embryonic heart rate appeared adequate, with strong and regular cardiac contractions.


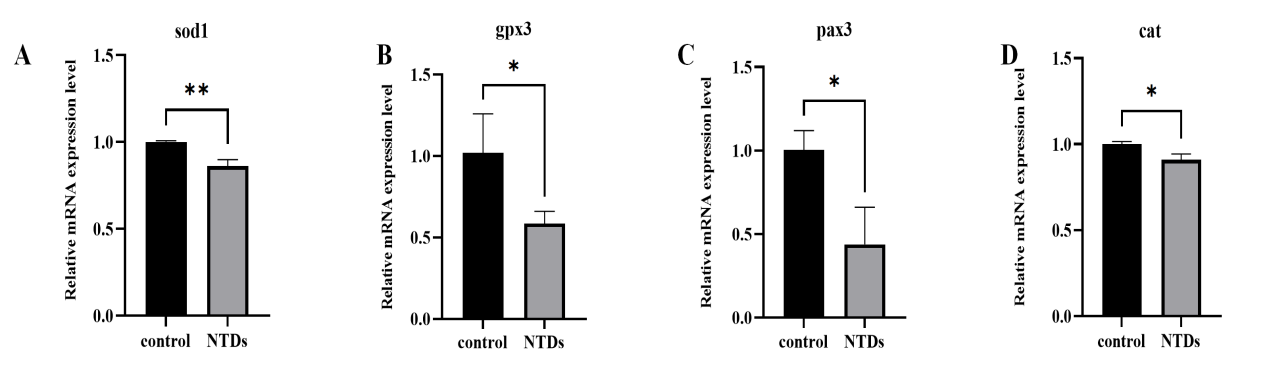


**Supplementary Figure S1. DTG exposure alters the mRNA expression of *Sod1*, *Gpx3*, *Pax3*, and *Cat* in mouse embryos** (A−D) The relative mRNA expression levels of antioxidant genes (*Sod1*, *Gpx3*, *Pax3*, and *Cat*) are determined in the control and NTD groups. Significant downregulation was observed in the NTD group, with *sod1* showing a profound decrease (***P* < 0.01), and *Gpx3*, *Pax3*, and *Cat* also presented reduced expression levels (**P* < 0.05).
